# Serious issues with cryo-EM structures of human prothrombinase

**DOI:** 10.1098/rsob.240193

**Published:** 2025-01-22

**Authors:** James A. Huntington, Alexandre Faille, Fatma Isik Ustok

**Affiliations:** ^1^ Department of Haematology, Cambridge Institute for Medical Research, University of Cambridge, The Keith Peters Building, Hills Road, Cambridge CB2 0XY, UK

**Keywords:** blood, cryo-EM, haemostasis

## Abstract

Thrombin is generated from prothrombin through sequential cleavage at two sites by the enzyme complex prothrombinase, composed of a serine protease, factor (f) Xa and a cofactor, fVa, on phospholipid membranes. In a recent paper published in *Blood*, Ruben *et al*. (Ruben *et al*. 2022 *Blood* **139**, 3463–3473 (doi:10.1182/blood.2022015807)) reported a major breakthrough in the field: the cryogenic electron microscopy structures of human prothrombinase on nanodiscs at 5.5 Å resolution (7TPQ) and of a catalytically inert human prothrombinase with its substrate prothrombin in the absence of any membrane at 4.1 Å resolution (7TPP). As is the norm in structural biology, the original paper was reviewed without access to the coordinates and maps, and it was therefore not possible for referees to assess the validity of the structures or their interpretations. In this article, we provide a post hoc analysis of the quality of the reported coordinates and maps, and look closely at the claimed intermolecular contacts on which the supposed breakthrough depends. We demonstrate that the work is deeply flawed, with not a single claimed intermolecular contact supported by the map, and conclude that the two reported structures do not contain any useful information regarding the assembly or function of the prothrombinase complex.

**Figure:**
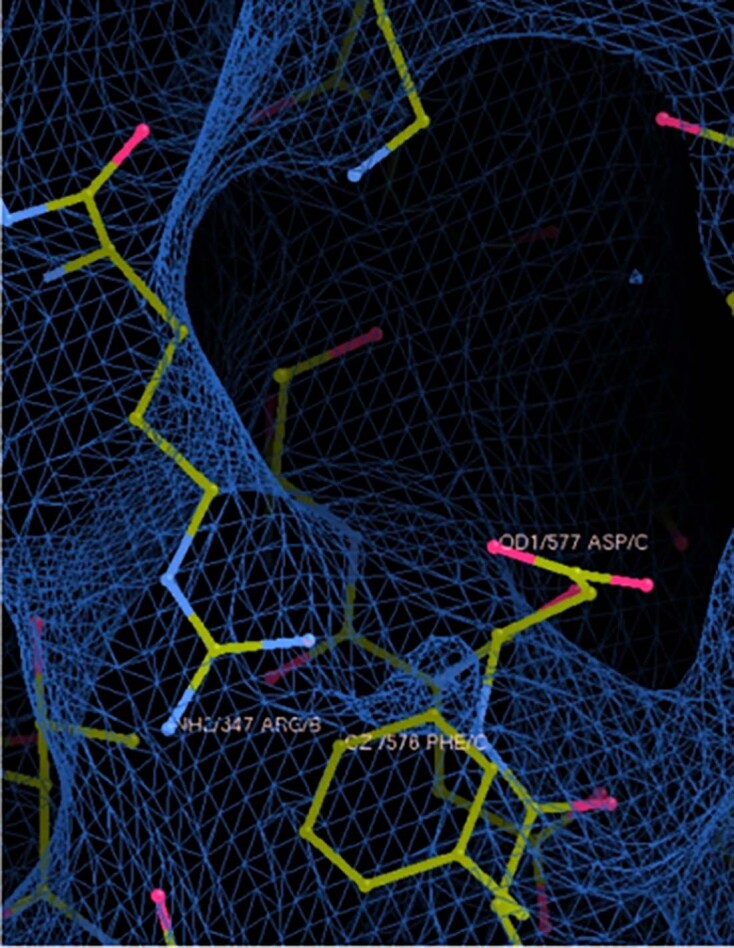

## 1. Introduction

**Figure 1.**
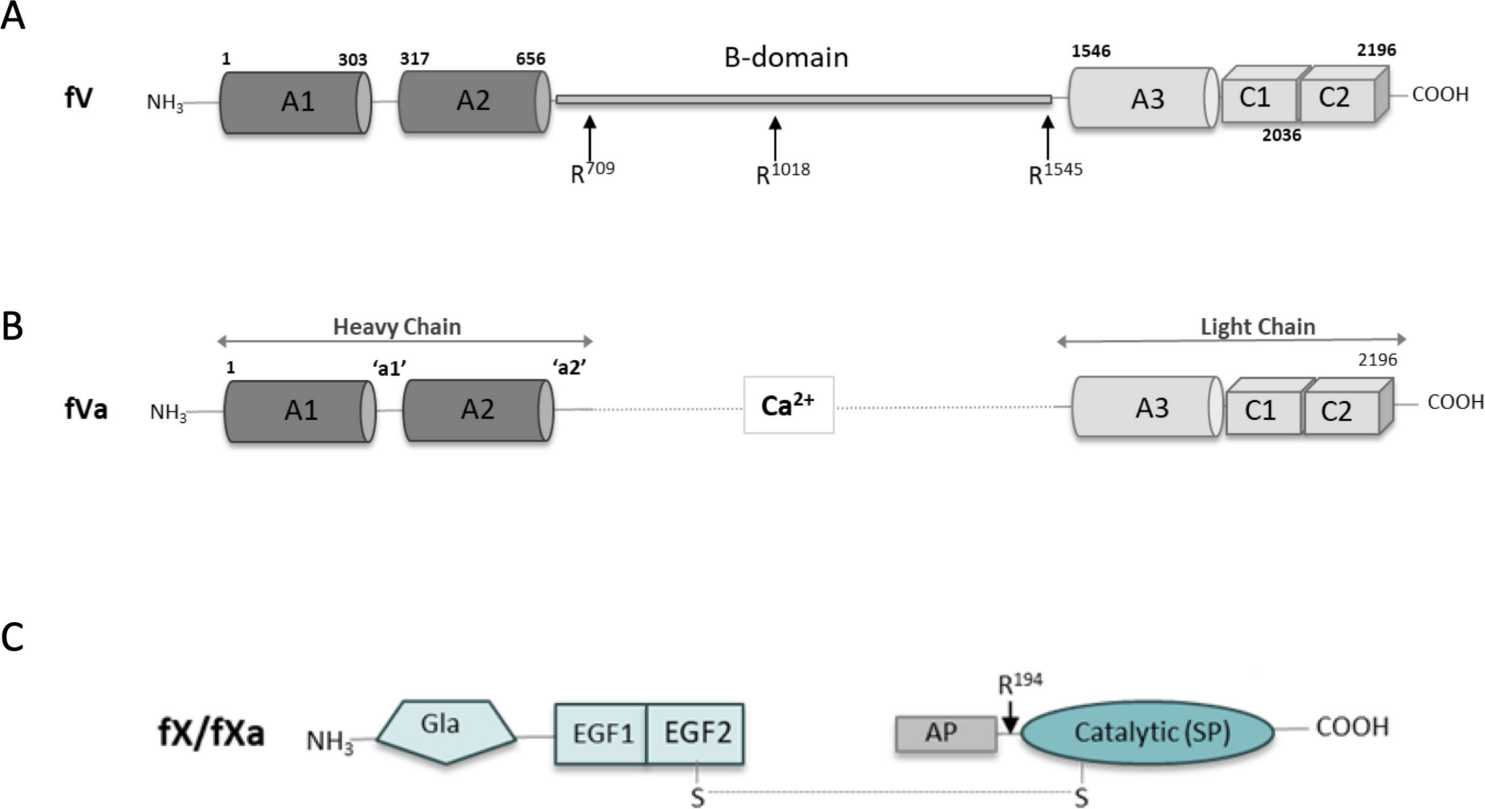
Schematics of fV/Va and fX/Xa. (A) fV is composed of 3 A domains and 2 C domains. A2 and A3 are linked by an unstructured region containing the B-domain. FV is converted to fVa by excision of the B-domain through cleavage at three sites (indicated). (B) The remaining stub of the linker is called the a2-loop and contains an acidic stretch important for assembly and function of prothrombinase. (C) fX is a two-chain zymogen that is activated to fXa through cleavage of the activation peptide (AP). It contains an N-terminal Gla-domain, two EGF-like domains and a catalytic domain (also known as the serine protease [SP] domain).

Thrombin is the effector enzyme in haemostasis; it activates platelets and converts fibrinogen into a fibrin mesh, the two components of a blood clot [1]. It is activated from its zymogen precursor prothrombin in the final step of the blood coagulation cascade by an enzyme complex known as prothrombinase. Prothrombinase is composed of a cofactor, factor (f) Va, and a serine protease, fXa, assembled on negatively charged phospholipid membrane surfaces that contain phosphatidylserine (PS), such as activated platelets [2]. The pro-cofactor fV is a single-chain protein composed of three A domains, two membrane-anchoring C domains and an unstructured and highly glycosylated B-domain that connects the A2 and A3 domains (figure 1A) [3]. Activation of fV to fVa is conferred through excision of the B-domain by thrombin and is thought to expose the fXa binding site [4], known to involve the A2 domain (figure 1B) [5]. Factor X is a two-chain zymogen (figure 1C) composed of an N-terminal membrane-anchoring gamma-carboxyglutamic acid (Gla) domain, two epidermal growth factor (EGF)-like domains and a chymotrypsin family protease catalytic domain (Cat) and is converted to the active serine protease fXa by cleavage of its activation peptide [6]. Factors Va and Xa interact with low affinity and generate thrombin inefficiently in the absence of PS-rich membrane surfaces. However, in the presence of negatively charged phospholipids, prothrombinase assembles with a Kd in the nanomolar range and processes prothrombin five orders of magnitude faster [7]. The dependence on a membrane interaction for assembly and function of prothrombinase is so absolute that some in the field suggest that Ca^2+^ (required for Gla-domain interaction with the membrane) and the membrane itself are additional components of prothrombinase [8]. A PS-rich membrane surface is, therefore, a key regulator of haemostasis since cells exposed to blood do not normally express PS on their outer leaflets unless damaged or activated and because assembly of both the intrinsic Xase complex (fVIIIa/fIXa) and prothrombinase absolutely depend upon its presence [9,10].

**Figure 2.**
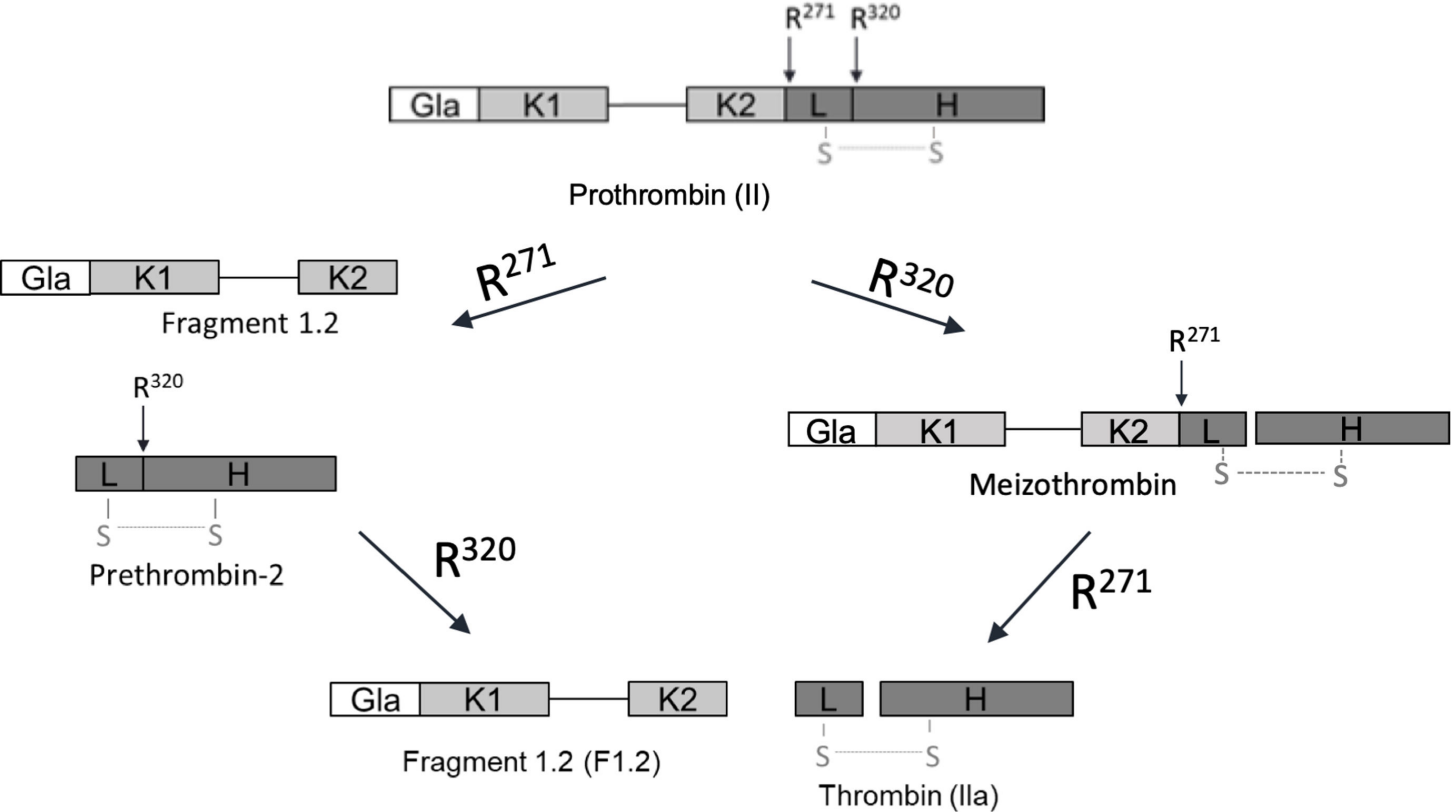
Schematic of prothrombin and its two processing pathways to thrombin. Prothrombin is a single-chain zymogen composed of a Gla-domain, two Kringle domains and a Cat domain, composed of a light (L) and a heavy (H) chain. Gla–K1 and K2–Cat are separated by a long flexible linker. Cleavage at both R271 and R320 is required to produce active thrombin. In the absence of fully assembled prothrombinase**,** processing occurs via the R271 route, whereas prothrombinase assembled on membranes processes exclusively down the R320 (meizothrombin) route.

In addition to increasing the rate of thrombin formation, the membrane surface also changes the order of prothrombin cleavage [11]. Prothrombin is a single-chain zymogen composed of an N-terminal Gla domain, two Kringle domains (K1 and K2) and a Cat domain (composed of a light [L] and a heavy [H] chain) [12]. It is converted to thrombin through cleavage at two sites, Arg271 and Arg320 (figure 2). Factor Xa alone, or in the presence of fVa but without membranes, first cleaves at Arg271, between the K2 and Cat domains, to sever fragment F1.2 (Gla–K1–K2) from prethrombin-2 (Pre-2; the zymogen form of the Cat domain). Much slower cleavage of Arg320, between the L and H chains, converts inactive Pre-2 into active thrombin. However, in the presence of fVa and a PS-rich membrane surface, fXa cleaves prothrombin first at Arg320 to produce the active intermediate meizothrombin, followed by Arg271 to release thrombin from the membrane-bound F1.2 fragment [11]. This order of cleavage makes physiological sense since initial cleavage at Arg271 risks releasing Pre-2 into the circulation before activation can be achieved.

A recent paper by Ruben et al. published in *Blood* [13] reports on cryogenic electron microscopy (cryo-EM) structures of human prothrombinase on nanodiscs and human prothrombinase bound to prothrombin in the absence of membranes. This work suffers from several fundamental flaws that call into question the veracity of the structures and their interpretation. In this manuscript, we discuss the methods, quality of the maps and unusual features of the deposited coordinates. We conclude that there is no useful information in the deposited structures 7TPQ or 7TPP and that all of the purported intermolecular contacts described in the paper published in *Blood* are unsupported by the maps and therefore unlikely to be correct.

## 2. Results

### 2.1. TPQ and 7TPP are identical

Ruben *et al*. [13] report two cryo-EM structures: fVa–fXa on nanodiscs at 5.3 Å resolution (7TPQ, EMD-26061) and fVa–fXa-prothrombin without membranes at ‘near atomic resolution’ of 4.1 Å (7TPP, EMD-26060). Models used for docking included a 3.3 Å cryo-EM structure of fV (7KVE) [14], a 6 Å crystal structure of ‘closed’ prothrombin (6BJR) [15] and a des-Gla version of fXa (1XKB) [16], with Gla-domain structures from 6BJR and 5EDM [17] used to build a model of full-length fXa. These coordinates were then fitted into the map of 7TPP (EMD-26060) and subjected to one round of real-space refinement in Phenix [18]. No information was provided on the type of restraints used, and there is no mention of local resolution. The resulting complex of fVa–fXa was then fit into the lower resolution map of prothrombinase on nanodiscs (EMD-26061) and then subjected to one round of real-space refinement. The deposited coordinates of 7TPQ are therefore identical to the prothrombinase component of 7TPP, save for one extra round of real-space refinement once placed into the map of 7TPQ. Because 7TPQ is identical to 7TPP and of significantly lower reported resolution, it is excluded from further analysis.

### 2.2. Grid conditions for 7tpp

It is highly unlikely that the reported grid conditions could have produced sufficient numbers of particles for structure determination. As mentioned in §1, prothrombinase assembly requires negatively charged phospholipid membranes. It is for this reason that nanodiscs were used to make grids of the prothrombinase complex in 7TPQ. However, for some reason, no surface, phospholipid or other support was used to make grids of the ternary complex of prothrombinase with prothrombin, nor was cross-linking employed (although it states in the methods section that it was attempted). The dissociation constant for the two components of human prothrombinase (fVa and fXa) in the absence of membranes is in the low micromolar range [19]. Grids were reportedly loaded with a solution containing a total protein concentration of 0.01 mg ml^−1^, with a 2:1:2 molar ratio of prothrombin:fVa:fXa (72, 168, 46 kDa, respectively). The concentration of fVa was therefore 25 nM, and the other two proteins were at 50 nM. A Kd of 1 µM should yield a concentration of prothrombinase of 1.2 nM, equivalent to 7.17 × 10^14^ particles per litre. They used Quantifoil 2/2 holey carbon grids with 2 μm diameter holes of 20 nm depth, with a volume of 0.063 μm^3^ (equivalent to 6.3 × 10^–17^ l). One would therefore expect to see 0.045 particles of complex per hole, requiring data collection from 100 entire holes to find about five particles of complex. It is reported that 330 317 particles were used to create the map. This would require the analysis of 7.3 million holes, which is impossible. It is unlikely the grid could have increased the local concentration sufficiently to overcome this issue, and, since the grids used did not contain a supporting carbon layer, there could not be a membrane-like effect to aid complex formation. Concentration at the air–water interface is possible, but not to an extent that would meaningfully alter the above conclusion, and generally particles are damaged by proximity to the interface. The optimal particle density for cryo-EM [20] is between 20 and 500 particles per μm^2^. The expected particle density for the conditions reported for 7TPP, assuming 100% complex formation, is only 0.3 per μm^2^. Because it is so surprising that this grid condition would even be attempted, perhaps a higher concentration was actually used.

**Figure 3.**
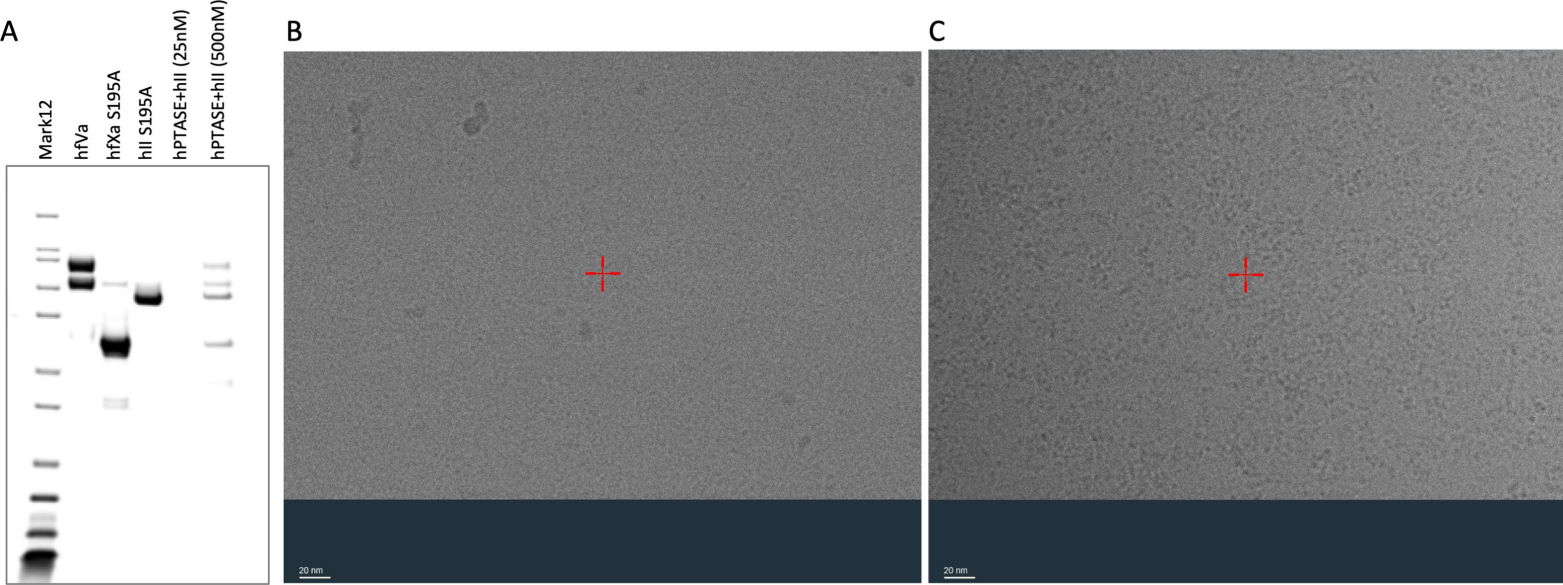
Repeat of grid conditions reported by Ruben *et al*. for 7TPP. (A) SDS gel of recombinant human fVa, S915A fXa and S195A prothrombin. The last two lanes are from the mixture at the 2:1:2 ratio used for making the grids shown in panels B and C, with the fVa concentration indicated. (B) Representative image of the 0.01 mg ml^−1^ total protein concentration condition reported for 7TPP and (C) of 0.2 mg ml^−1^ total protein concentration (scale shown bottom left for each).

We tested whether the reported grid conditions could yield visible particles by repeating their experiment with the same concentrations of fVa, S195A fXa and prothrombin (all recombinant, and prothrombin was mutated to S195A to prevent auto-activation) using the same grids, blotting force and time. Duplicate grids were vitrified using a Vitrobot Mark IV and imaged on a Titan Krios 300kV microscope. An SDS gel of the material used and a representative micrograph are shown in figure 3. As predicted, the grids with conditions identical to those reported for 7TPP appear empty, with no sign of even the relatively large and distinctive shape of fVa. Grids at a 20-fold higher protein concentration (0.2 mg ml^−1^) produced images where distinct particles could not be resolved by eye, resembling ‘soup’ (figure 3C), suggesting that the proteins were not forming a complex, as predicted from a Kd in the micromolar range.

### 2.3. Quality of the 7TPP map (EMD-26060)

**Figure 4.**
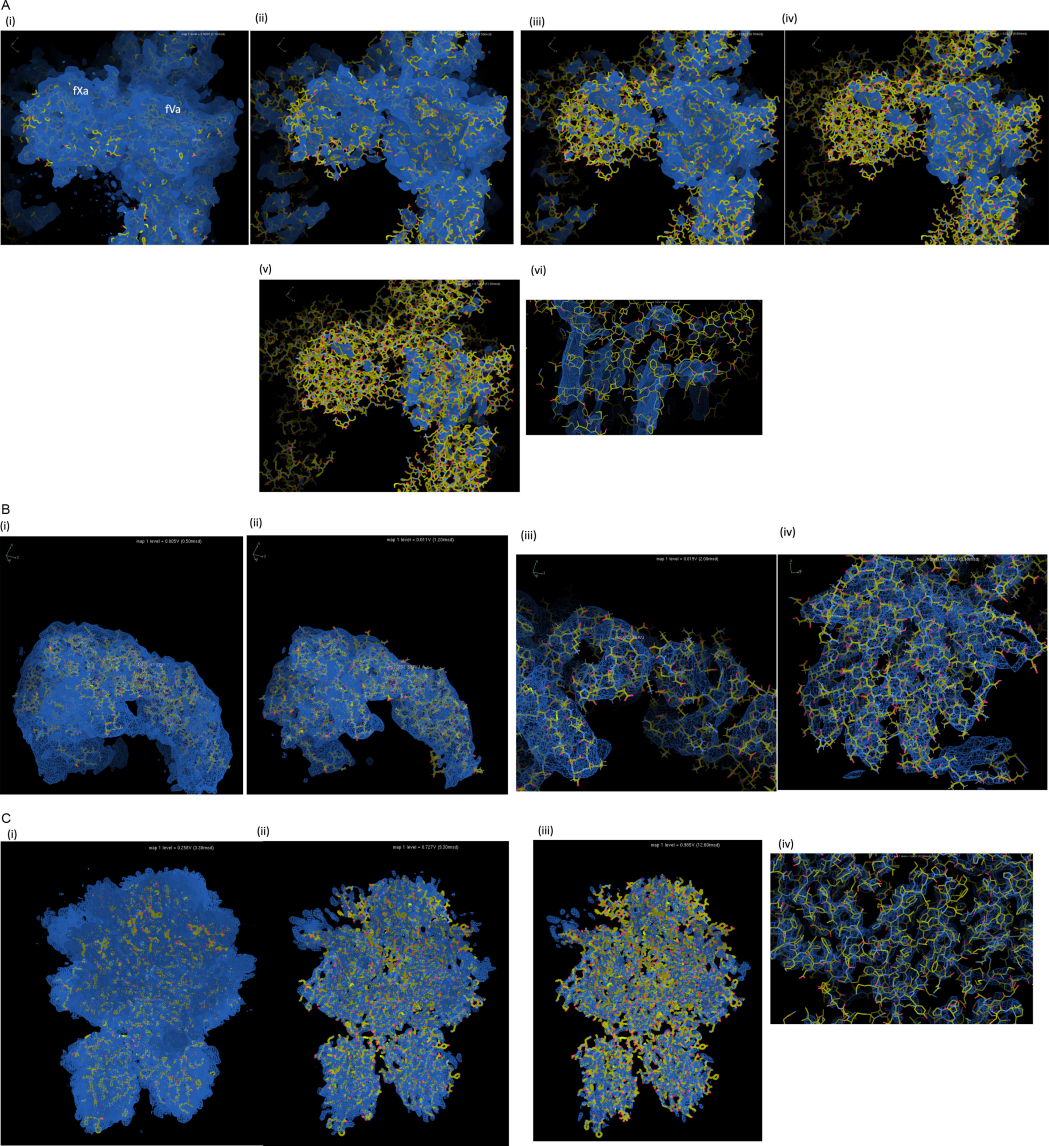
Maps from cryo-EM structures at increasing contour levels. (A) Six panels (i–vi) of 7TPP focusing on fVa (right part of each image) and fXa (left part). Final panel (vi) is a close-up of panel (v). (B) Four panels (i–iv) of an unrelated small asymmetrical protein with a cryo-EM map of 4.1 Å resolution (7R9K). (C) Four panels (i–iv) of 7KVE, a 3.3 Å structure of fV and its corresponding cryo-EM map. Screenshots taken from Coot [22], and contour levels are indicated in the upper right hand side of each panel.

The map claims to be 4.1 Å and of ‘near atomic resolution’, but in no region does the deposited map resemble a typical 4.1 Å cryo-EM structure. One can also simulate a 4.1 Å resolution map in Chimera [21], and doing so using the coordinates of 7TPP results in a map bearing little resemblance to the deposited map for any region. No information regarding local resolution estimates was provided in the manuscript or deposition, which is unusual and prevents analysis of the relative certainty of atomic position. Increasing the contour level of the map, however, similarly highlights the areas and elements in the structure with the highest level of confidence. For EMD-26060, this exercise yields a non-sensical striated pattern with no corresponding protein structure (figure 4A). An unrelated, randomly selected cryo-EM structure at 4.1 Å resolution, 7R9K [23], is shown as an example of a map generated from small asymmetrical protein particles and the effect of increasing contour levels (figure 4B). An example closer to home is 7KVE, a cryo-EM structure of fV at 3.3 Å, also reported by the Di Cera group [14]. The difference in the map when compared to 7TPP is striking and illustrates how the map of a beta-sheet protein should behave when increasing the contour level (figure 4C). The information content of the map EMD-26060 is therefore not what would be expected based on the reported resolution of 4.1 Å.

### 2.4. 7TPP does not represent a productive enzyme-substrate complex

7TPP is meant to be prothrombinase bound to its substrate prothrombin. Since the fXa used was inert, having the catalytic Ser residue substituted for Ala, one would expect the active site to be occupied by the substrate. It is therefore quite unusual that the active site of fXa is unoccupied, with neither Arg271 nor Arg320 of prothrombin interacting. However, Arg320 is reported to be near the active site (7.1 Å) with the ‘sequestering of R271 against D697’ (of fVa) ‘directing R320 toward the active site of fXa’. This is also surprising since it is well known that in the absence of a membrane surface, fXa in the presence of fVa preferentially cleaves prothrombin down the Pre-2 route (initial cleavage of Arg271), so one would reasonably expect to see Arg271 in the active-site S1 pocket of fXa (S1 is the primary specificity pocket that binds to the Arg side chain). In any case, the absence of either Arg271 or Arg320 from the active site of fXa means that 7TPP does not represent a productive complex of prothrombinase with prothrombin.

### 2.5. The unusual conformation of fXa

**Figure 5.**
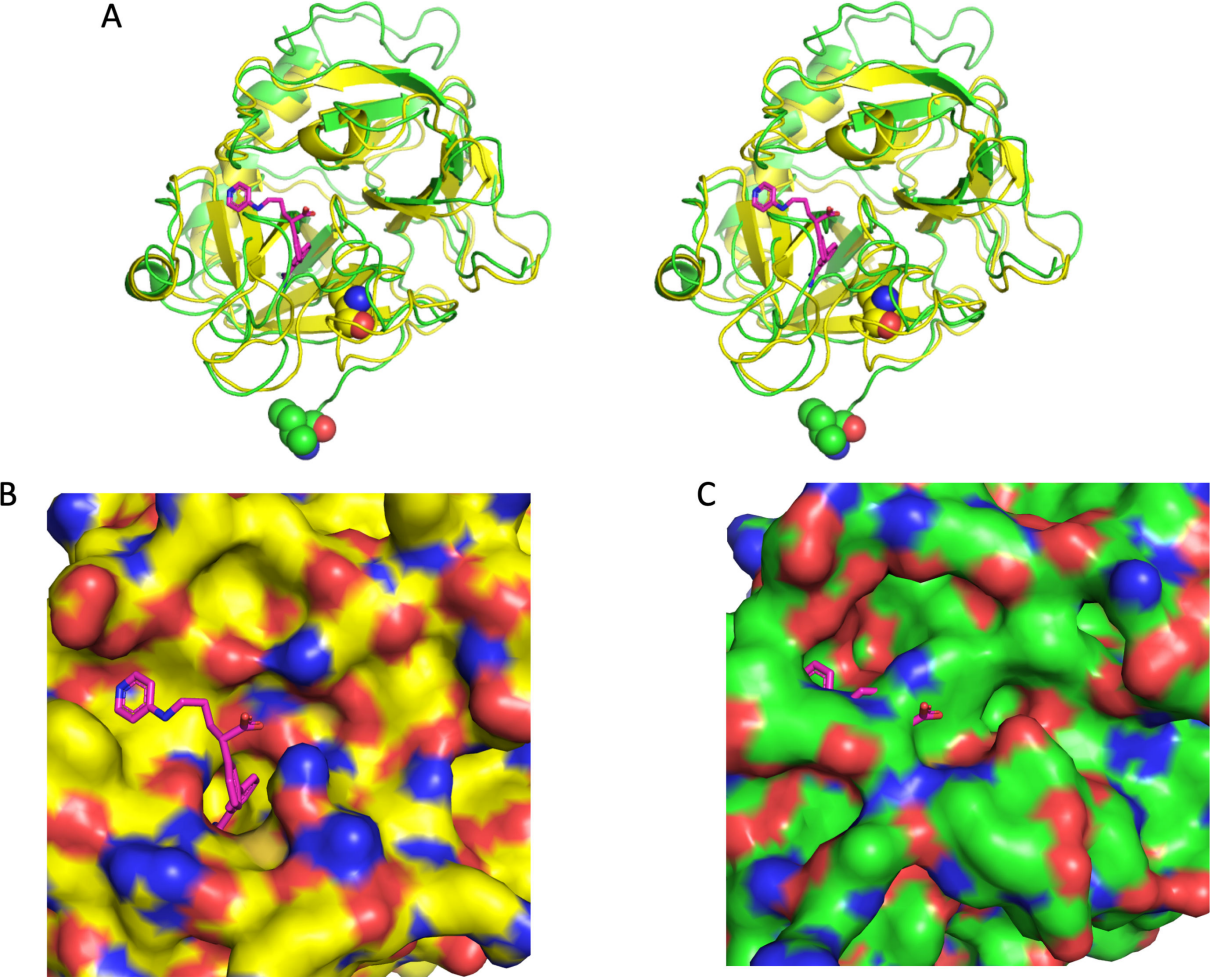
Factor Xa Cat domain structures from 1XKB (yellow) and 7TPP (green). (A) Stereo view of cartoon representations of the superimposed Cat domains from 1XKB and 7TPP in the standard orientation. The N-terminal Ile16 residue is shown as balls, and the inhibitor from 1XKB is shown as magenta sticks. (B) Surface representation of the active site of 1XKB with inhibitor bound. The inhibitor fits nicely into an open active-site cleft, buries deeply into the S1 pocket and partially occupies the S4 pocket. (C) The same representation of the Cat domain of fXa from 7TPP shows a very different topology and a closed-off active site (the inhibitor from 1XKB is shown to illustrate the incompatibility of the deposited structure with inhibitor or substrate binding). Figures made using PyMOL (v.2.5.2 Schrödinger, LLC).

The structure of fXa in 7TPP is distorted and not in an active conformation. This is surprising since the model of full-length fXa placed in the map was based on 1XKB, Gla-domainless fXa bound to an active-site inhibitor [16] and only one round of real-space refinement was reportedly conducted. Indeed, the structure has deviated greatly from the starting model, with a Cα root mean square deviation (RMSD) of 2.83 Å for 235 residues in the Cat domain. A deviation of note is that the N-terminus of the heavy chain (the activation loop) is not docked in the activation pocket and making a salt bridge to Asp194, as expected for an active serine protease [24], but rather pointing out into space (figure 5A). Indeed, the conformation of the catalytic loop from 185 to 195 (chymotrypsin template numbering) bears little resemblance to any deposited structure of fXa and is inconsistent with catalytic activity (e.g. there is no oxyanion hole). In addition, the side chain of Trp215 is flipped, partially blocking the active site, and the 191−220 disulfide has moved into the S1 pocket precluding substrate or inhibitor binding (figure 5B versus 5C). It appears that the fXa component of 7TPP is actually the zymogen fX. This is all very mysterious, since a single round of real-space refinement, even into a meaningless map, would not result in such dramatic and unlikely conformational changes, especially with restraints weighted for a low-resolution map. This is yet another reason to conclude that 7TPP does not represent the structure of an active prothrombinase complex.

### 2.6. Paucity and unusual nature of intermolecular contacts

**Figure 6.**
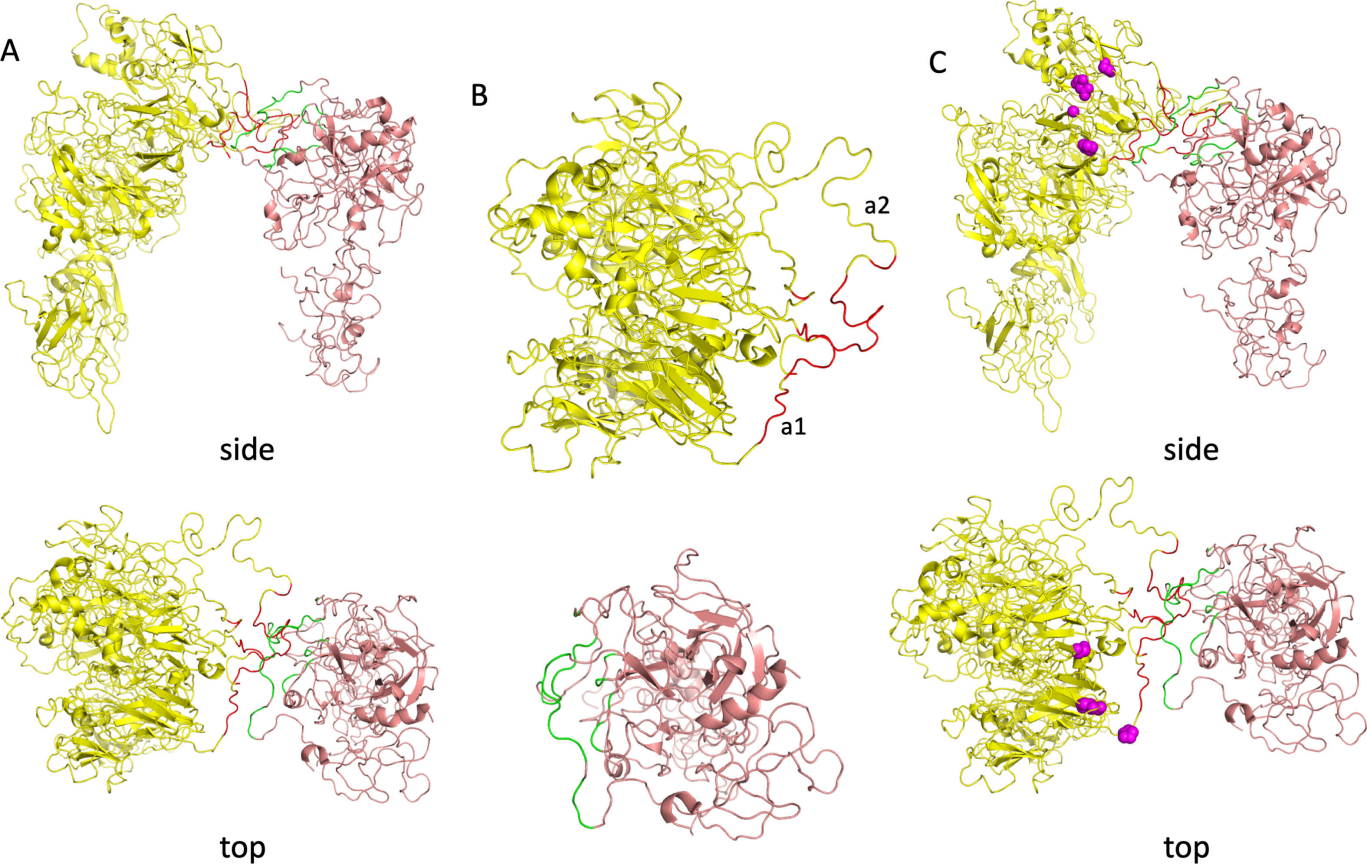
Illustration of the unusual contact between fVa (yellow, with red for contacts) and prothrombin (salmon, with green for contacts). (A) Side and top view of the interaction. (B) Individual top views of fVa and fXa, coloured as before. (C) Side and top view as in *a*, with magenta balls illustrating the positions that when PEGylated adversely affect prothrombin processing by pseutarin C. Figures made using PyMOL (v.2.5.2 Schrödinger, LLC).

Another strange feature of 7TPP relates to the paucity of intermolecular contacts and the central involvement of flexible loops at the interfaces. This is particularly troubling for the fVa–prothrombin interface. Prothrombin is barely touching fVa and appears to angle out to avoid making any further contact with other domains (figure 6). Indeed, the distance between the C domains of fVa and the Gla domain of prothrombin is about 60 Å. The contacting fVa regions lie exclusively on the flexible a1- and a2-loops (the a1-loop connects the A1 and A2 domains), and on a linker between two beta strands (residues 503 and 505), with nearly two-thirds (63%) of the buried surface area on fVa involving the a2-loop. Similarly, on prothrombin, residues from 258−271 and 305−316 make contact, and both stretches belong to unstructured loops. Looking from above it appears that the two molecules are touching outstretched fingers from a distance (figure 6A, lower panel, and separated in panel B). This is not how proteins typically interact and could not possibly account for a 300 000-fold increase in the rate of prothrombin processing for assembled prothrombinase, which must be primarily attributable to the interaction between fVa and prothrombin. Furthermore, as will become evident when discussing the interactions themselves in a later section, the interface is not supported by experimental data (i.e. the map). The approach of prothrombin towards the front face of fVa is also inconsistent with the density of consensus N-linked glycosylation sites (from the sequences of fV from 37 species) that would prevent protein–protein interactions [25] and with a recent PEGylation study [26] that demonstrated important prothrombin contacts on pseutarin C (a homologous snake venom prothrombinase) within the A1 domain (residues 276 and 302) and with the ‘top’ of the A2 domain (residues 441 and 455; contact residues shown as magenta balls in figure 6C). This is another indication that 7TPP does not represent a productive complex between prothrombinase and prothrombin.

### 2.7. The a2-loop

The a2-loop is the N-terminal portion of the B-domain, a long unstructured stretch that links the A2 and A3 domains in fV (see figure 1). When fV is activated by thrombin to form fVa, the a2-loop is all that remains of the B-domain and protrudes from the C-terminal end of the A2 domain after Cys656 (residues 657−709 constitute the a2-loop). It has a conserved acidic stretch typically containing at least one sulfated tyrosine that has been shown to be involved in prothrombinase assembly and function [27,28]. In 7TPP, the a2-loop is modelled across the face of fVa and makes the bulk of the contacts with both fXa (Cat domain) and prothrombin (79 and 63% of buried fVa surface area with each, respectively). However, the placement of the a2-loop is not supported by the map and appears not to have been modelled independently, but taken from a previously published cryo-EM structure of fV (7KVE).

**Figure 7.**
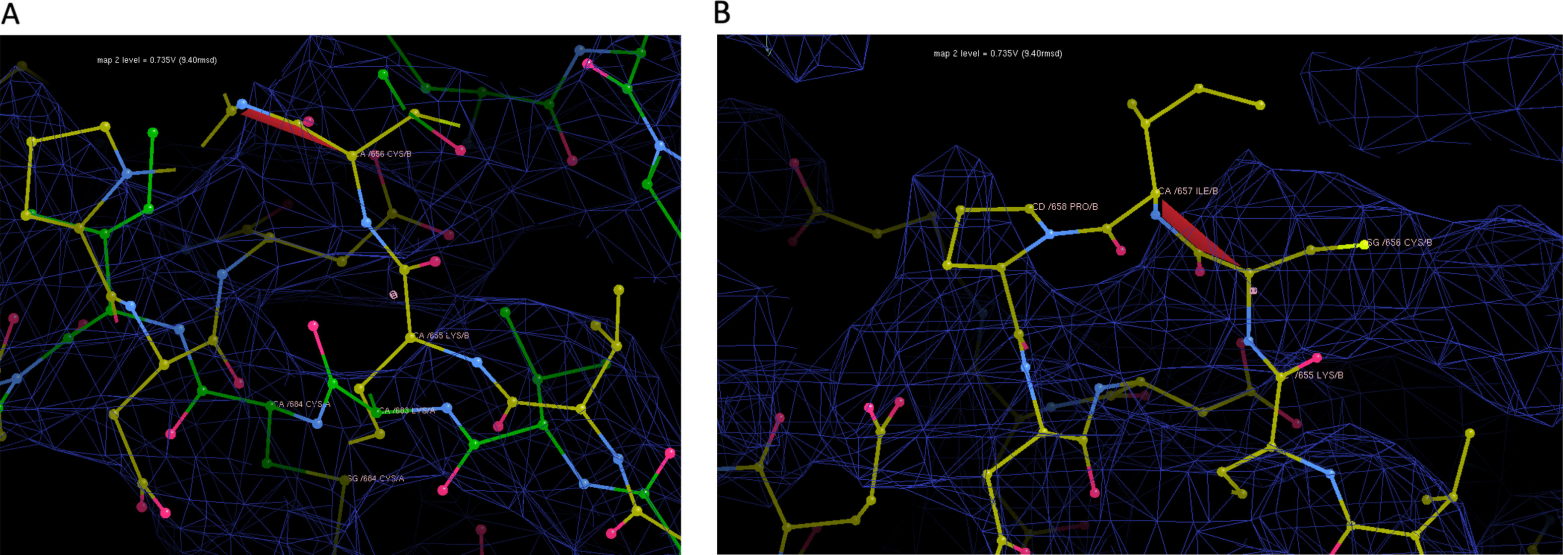
Screenshots from Coot illustrating the breaking of the Cys575–Cys656 disulfide bond and other structural abnormalities in 7KVE. 7KVE is shown with yellow carbon atoms and the Alphafold fV model with green carbon atoms. The map (EMD−23048), contoured as indicated, is shown as blue wire. (A) The correct disulfide bond of the Alphafold model is partially shown in green at the bottom (numbering is different, but Cys656 is depicted). Cys656 has moved towards the top of the image in 7KVE, taking it and the following residues out of the map. (B) The unusual and incorrect placement of Cys656 in 7KVE also produces a *cis*-peptide bond (shown in red) with Ile657.

The 3.3 Å structure of fV (7KVE, EMD-23048) previously published by the Di Cera group [14] has obvious and serious errors in the positioning of the a2-loop that was propagated to 7TPP. The a2-loop starts after Cys656, which is known to form a disulfide with the adjacent Cys575. However, in 7KVE, the disulfide bond between 575 and 656 is broken and Cys656 has moved approximately 10 Å, making disulfide bond formation impossible (figure 7). All other expected disulfide bonds are maintained in 7KVE. The movement of Cys656 is potentiated by a deviation in the position of Lys655, which jumps across a gap in the map. The Alphafold [29] model of fV (green in figure 7A) unsurprisingly preserves the disulfide bond and actually fits into the map. Since the map of 7KVE is consistent with the preservation of the disulfide, it must be assumed that the breaking of the disulfide bond and movement of the loop was somehow done by mistake. However, the direction of the a2-loop after Cys656 in 7KVE is influenced by the breaking of the 575−656 disulfide bond and a *cis*-peptide bond between Cys656 and Ile657 (figure 7B).

**Figure 8.**
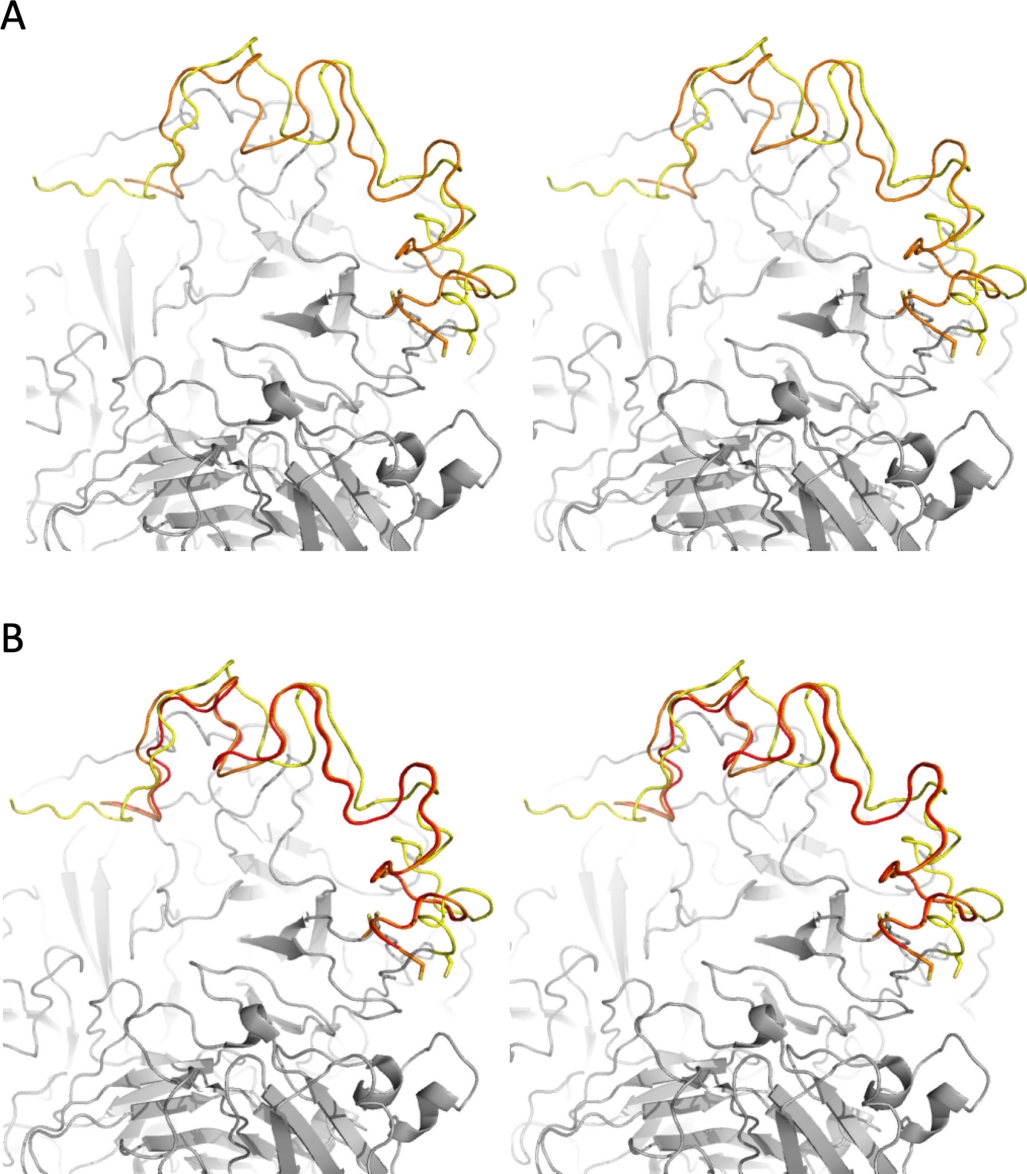
Stereo images illustrating that the conformation of a2-loops is conserved between 7KVE, 7TPP and 7TPQ. (A) The a2-loop is in the same conformation in 7KVE (yellow for a2-loop and grey for the rest of the structure) and 7TPP (orange), only slightly shifted. (B) The a2-loops of 7TPP (orange) and 7TPQ (red) are identical (a2-loop from 7KVE in yellow is shown for comparison). Figures made using PyMOL (v.2.5.2 Schrödinger, LLC).

The physically improbable positioning of the a2-loop in 7KVE is also not supported by the data, with residues 657−658, 662−664, 667, 670−672, 674−675 and 677−713 (end) all outside of the map (EMD-23048; see electronic supplementary material, figures). It is therefore highly unlikely that the conformation and position of the a2-loop in two ‘independent’ cryo-EM structures of fVa bound to other proteins (7TPQ and 7TPP) would be identical. However, 7KVE was the structure placed into the map of 7TPP, and the conformation of the a2-loop is largely preserved (54 main chain atom RMSD of 3.25 Å) with only a slight, 5 Å shift in position (measured at His682; figure 8A). The conformation of the a2-loop in 7TPQ is identical to 7TPP (RMSD 0.7 Å for 54 atoms and 0.206 Å for 41 atoms; figure 8B). The broken disulfide and *cis*-peptide bonds are also carried over from 7KVE.

Unsurprisingly, the position and conformation of the a2-loop in 7TPP are also not supported by its map (EMD-26060). Residues 657, 658 and 666−667 are outside of the map, and there is no continuous density from 690 to 709 (end; see electronic supplementary material, figures). In total, 24 out of 53 modelled a2-loop residues are not placed within the map. For those residues that are surrounded by map it is still impossible to make any statements concerning their conformation or position, due to the utter lack of features of the map at any contour level (i.e. the map is a blob). The inescapable conclusion is that the position of the a2-loop in all three structures is grossly incorrect and completely wrong with respect to any details (i.e. position of side chains). It is therefore impossible to make any statements concerning a2-loop residues in mediating intermolecular contacts with either fXa or prothrombin in 7TPP. And yet, the bulk of the reported interactions that fVa makes with fXa (Cat domain) and prothrombin involves the a2-loop (79 and 63%, of buried surface area, respectively). Each individual claimed interaction, listed in Table 2 of the paper describing 7TPP [13], is discussed in the following sections, with a2-loop residues indicated by an asterisk. Distance cut-offs for hydrogen bonds, salt bridges and hydrophobic interactions used in this analysis are 3.5, 4 and 5 Å, respectively [30–32] .

### 2.8. fVa–fXa interactions (figure 9)

**Figure 9.**
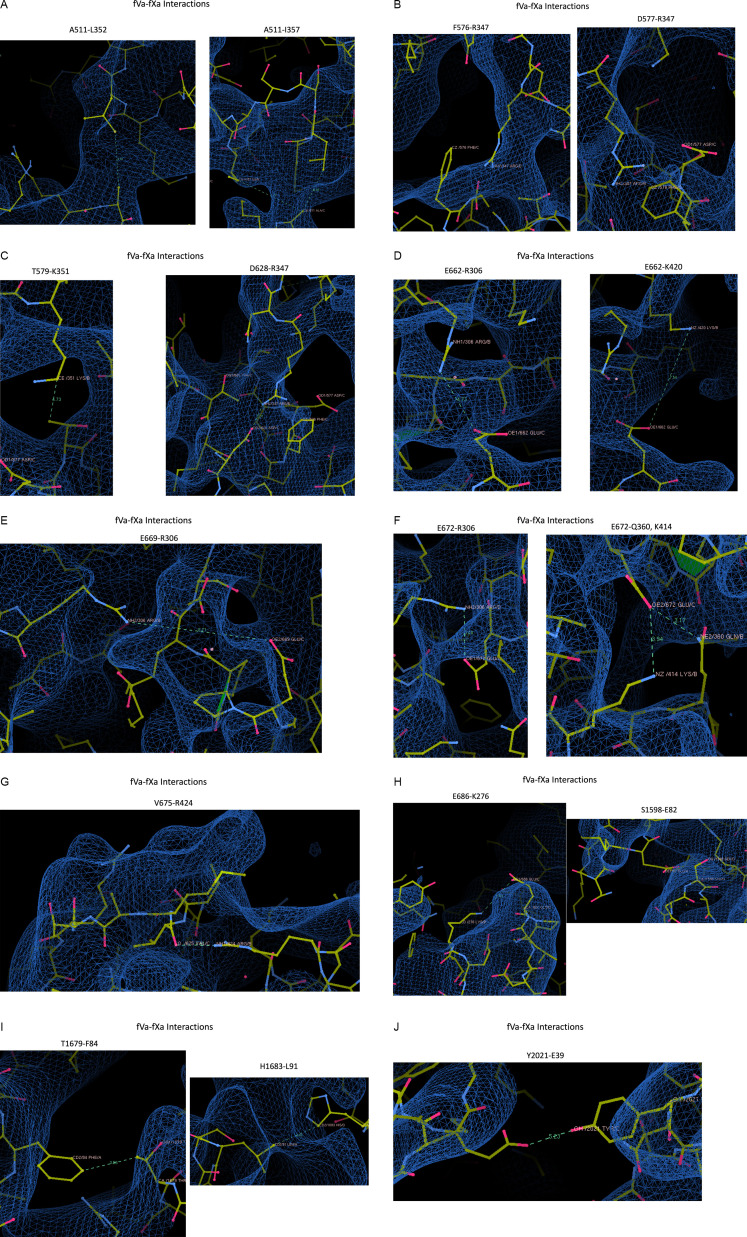
Screenshots from Coot of the fVa–fXa interactions listed in Table 2 of Ruben *et al*.'s paper [13], with the map contoured at 0.033V. Seventeen panels are given illustrating each of the claimed interactions (indicated above each panel). Contacts are indicated by dashed lines with distances.

*Hydrophobic interaction between A511–L352*: The closest distance between any two atoms is 4.38 Å. The contact involves the only atom of the side chain of L352 that is in the map, with the rest of the sidechain found outside of the map.

*Interaction between A511–I357*: The closest atoms are 4.98 Å apart, N of A511 and CG1 of I357. The type of atoms and the distance are incompatible with a favourable interaction, and CG1 of I357 is not in the map.

*Pi stack between F576–R347*: The distance (3.39 Å) is consistent with a favourable interaction, but neither sidechain is in the map.

*Salt bridge between D577–R347*: The distance (2.91 Å) is consistent with a salt bridge, but neither sidechain is in the map.

*Hydrophobic interaction between T579–K351*: The distance between CG of 579 and CE of 351 is 4.71 Å, but neither atom is in the map.

*Salt bridge between D628–R347*: The distance is too close (2.31 Å), so contacting atoms OD2 and NH2 actually clash. As before, the sidechain of R347 is not in the map.

E662*–R306 contact: The closest distance for any two atoms is between CG and NH2 (4.86 Å). This is not a favourable contact. Neither side chain is enclosed in the map, and both of the ‘interacting’ atoms are outside the map.

Salt bridge between E662*–K420: The closest distance between any two atoms is inconsistent with a contact (7.54 Å), and neither side chain is in the map.

Salt bridge between E669*–R306: The distance between the closest atoms is inconsistent with a contact (8.71 Å), and neither side chain is in the map. In addition, the direct line between the closest atoms runs through space occupied by OE2 from E662 and main chain O atoms from P671 and E672.

Salt bridge between E672*–R306: The distance is consistent with a salt bridge (3.48 Å), but neither side chain is in the map.

Hydrogen bond between E672*–Q360: The side chain of E672 is not in the map, and the entire amino acid Q360 is out of the map.

Salt bridge between E672*–K414: Both side chains are out of the map.

Hydrogen bond between main chain O of V675* and side chain of R424: The distance is consistent with a hydrogen bond, and all atoms are in the map. This is the only interaction reported in Table 2 that meets these two simple criteria. However, the map is of insufficient quality to have any confidence in the position of the atoms in the backbone of V675 or the side chain of R424.

Salt bridge between E686*–K276: The distance (5.63 Å) is inconsistent with an interaction, and neither side chain is in the map.

*S1598–E82*: No side-chain interaction is possible. A hydrogen bond is possible between N of S1598 and OE1 of E82 (2.81 Å); however, the entire residue of E82 is out of the map.

*Hydrophobic interaction between T1679–F84*: The closest contact is between CG2 of T1679 and CE2 of F84 (3.56 Å), but CG2 of T1679 and the entire side chain of F84 are not in the map.

*Hydrophobic contact between H1683–L91*: The closest atom on L91 to H1683 (3.46 Å) is out of map.

*Hydrogen bond between Y2021–E39*: The distance between OH of Y2021 and OE1 of E39 is inconsistent with a hydrogen bond (5.02 Å), and neither side chain is in the map.

### 2.9. Prothrombin-fXa interactions (figure 10)

**Figure 10.**
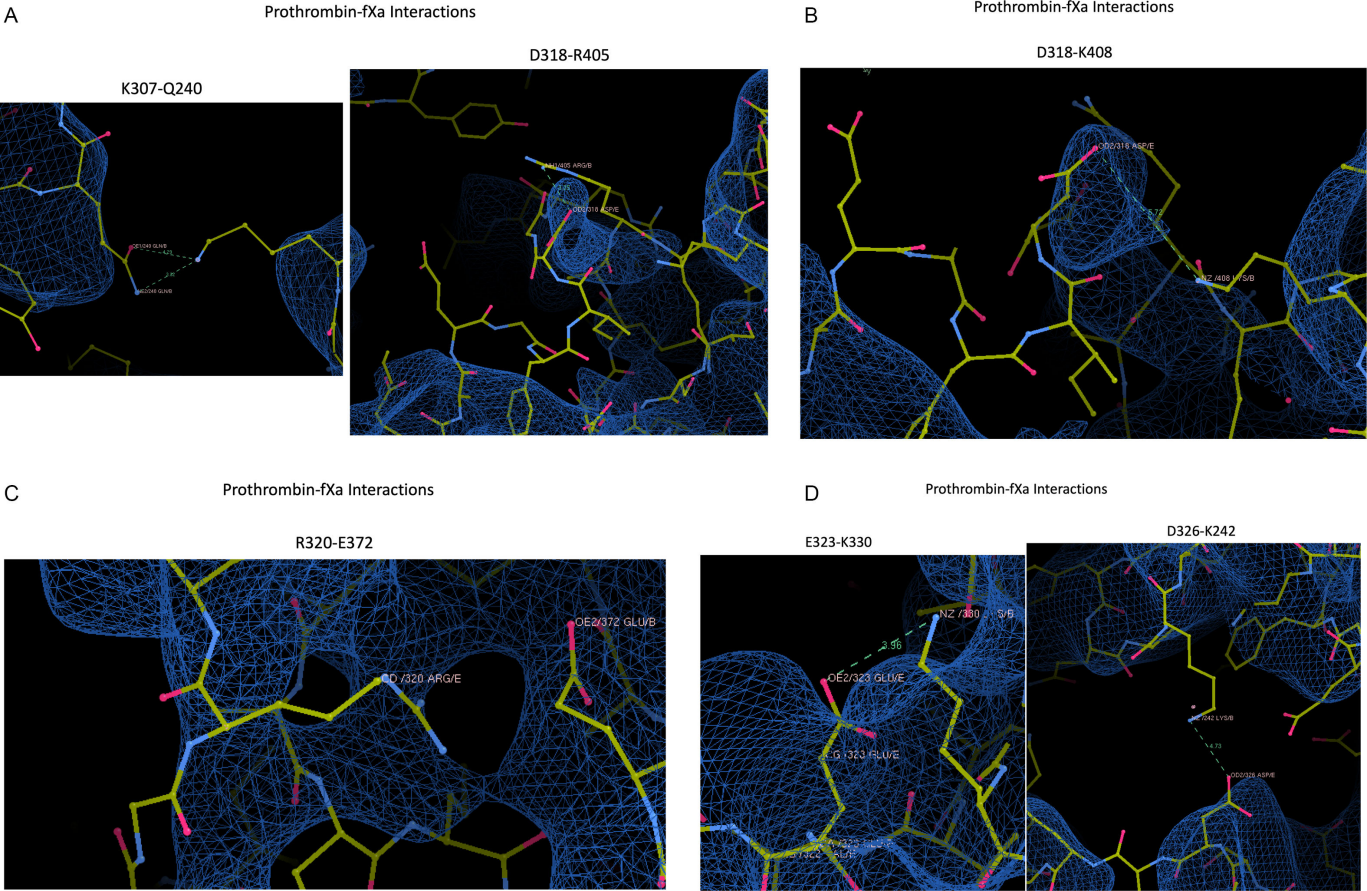
Screenshots from Coot of the prothrombin–fXa interactions listed in Table 2 of Ruben *et al*., with map contoured at 0.033V. Six panels are given illustrating each of the claimed interactions (indicated above each panel). Contacts are indicated by dashed lines with distances.

*Hydrogen bond between K307–Q240*: Neither side chain is in the map, and Q240 would need to flip to make the hydrogen bond (3.32 Å).

*Salt bridge between D318–R405*: No residue is in the map for prothrombin between 313 and 321, including D318. Nothing is in map for fXa from 396 to 406, including R405.

*Salt bridge between D318–K408*: The distance between side chain atoms (5.72 Å) is incompatible with a salt bridge. Residue D318 and the side chain of K408 are out of the map.

*Salt bridge between R320–E372*: The closest contact is between NH1 of R and CG of E is 3.17 Å, and these atoms are unable to make a salt bridge. The geometries and distances between the Ns of R320 and the Os of E372 are inconsistent with a salt bridge. R320 is not in the map, and nothing is in map for fXa from 371 to 379, including E372.

*Salt bridge between E323–K330*: The distance (3.96 Å) between ‘interacting’ atoms is consistent with a salt bridge, but neither side chain is in the map.

*Salt bridge between D326–K242*: The distance between ‘interacting’ atoms (4.73 Å) is inconsistent with a salt bridge. Residues 326−327 of prothrombin are not in the map, and residues 240−246 of fXa are not in the map.

### 2.10. Prothrombin–fVa interactions (figure 11)

**Figure 11.**
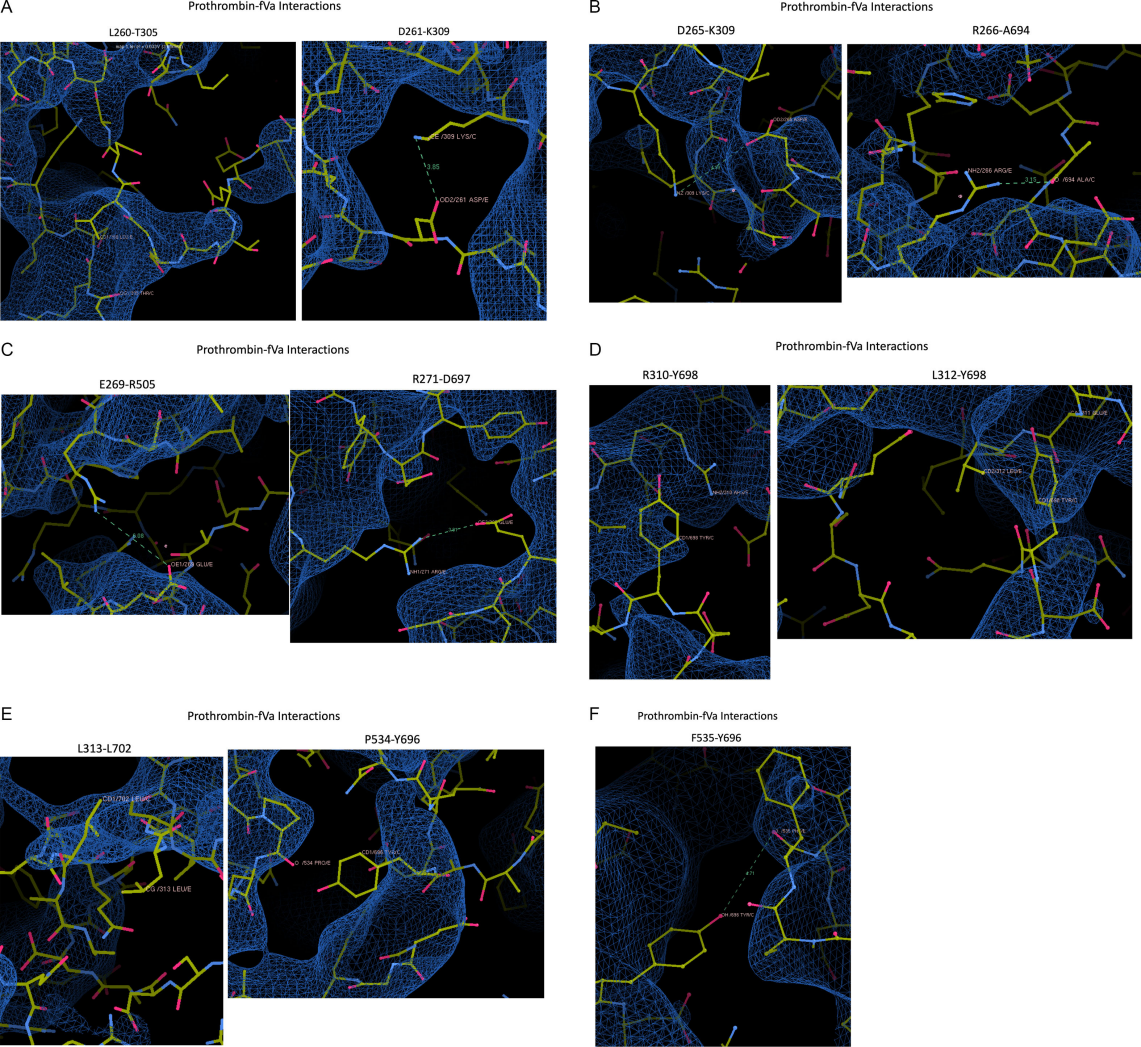
Screenshots from Coot of the prothrombin–fVa interactions listed in Table 2 of Ruben *et al*., with map contoured at 0.033V. Eleven panels are given illustrating each of the claimed interactions (indicated above each panel). Contacts are indicated by dashed lines with distances.

*Hydrophobic contact between L260–T305*: The closest two atoms are 4.75 Å apart, but residues 252−259 of prothrombin are not in the map and only two main chain atoms of L260 are in the map.

*Salt bridge between D261–K309*: Neither residue is in the map.

*Salt bridge between D265–K309*: The distance of 4.99 Å is inconsistent with a salt bridge. The side chain of D265 is not in the map, and the entire residue K309 is out of the map.

Hydrogen bond between R266–A694*: The side chain of R266 is not in the map. Residues 690−695 of fVa are not in the map.

*Salt bridge between E269–R50*5: The distance of 6.08 Å is inconsistent with a salt bridge, and the closest two atoms are not in the map.

Salt bridge between R271–D697*: The geometry is inconsistent with a salt bridge. The side chain of R271 is not in the map. Residues 697−709 of fVa are not in the map.

Pi stack between R310–Y698*: The final three atoms of the side chain of R310 are not in map. Residues 697−709 of fVa are not in the map.

Hydrophobic interaction between L312–Y698*: The side chain of L312 is not in the map. Residues 697−709 of fVa are not in the map.

Hydrophobic interaction between L313–L702*: Residues 313−321 of prothrombin are not in the map, and residues 697−709 of fVa are not in the map.

Hydrogen bond between P534–Y696*: The contacting atoms are out of the map (O from P534 and OH from Y696). The entire side chain of Y696 is not in the map.

Hydrogen bond between F535–Y696*: The distance between O of F535 and OH of Y696 is 4.71 Å, inconsistent with a hydrogen bond. F535 is not in the map, and the entire side chain of Y696 is not in the map.

## 3. Discussion

This is by no means an exhaustive critique of the manuscript by Ruben *et al*. or the structures described therein. Indeed, it appears that everything is wrong: the grid conditions; the structures placed in the maps (fVa and fXa were touched upon, but the modelled prothrombin is from a 6 Å crystal structure, and the Gla domains of prothrombin and fXa are both in an incorrect conformation); the claimed resolution; and the reported contacts. It is, however, abundantly clear from this limited analysis that the structure is of exceedingly low quality and that the described intermolecular contacts are not real.

We originally speculated that 7TPP might be a case of ‘Einstein from noise’ to rationalize the several pathologies in the map EMD-26060. These include, but are not limited to: a complete lack of features corresponding to secondary structural elements; a general blob-like topology inconsistent with the stated resolution, resembling a mask; a floating blob fit to prothrombin suggestive of an artefact caused by reference bias; a worrying lack of coalescence of map on ordered domains composed of beta sheets upon increasing of the contour level; the directional striation of the map when contour is increased, bearing no resemblance to the underlying structure; and half-maps that appeared to be nothing more than a concentration of noise inside of a mask. These pathologies are consistent with severe reference bias. When we were unable to observe any particles on grids made using the reported, exceedingly low, protein concentration of 0.01 mg ml^−1^, the conditions for Einstein from noise appeared to have been met. We contacted the corresponding author Enrico Di Cera to express our concerns and to ask for the deposition of the micrographs on the EMPIAR server. To our surprise and to his credit, the motion-corrected micrographs were deposited 10 days later and were released two weeks after that (28 July 2023). We analysed just over one-third of the deposited data and found that the micrographs contain actual particles and that the deposited map is not the result of Einstein from noise. Although it is now clear that the map was not generated from Gaussian noise, it is still of exceedingly poor quality and suffers from several pathologies, in part due to a severe orientation bias discovered when processing the deposited data. It is simply not possible to use such a low-quality map to draw conclusions about how the proteins that constitute the prothrombinase complex interact, beyond a rough placement of domains. The manuscript describing 7TPP amounts to a gross overinterpretation of the data.

This is particularly relevant to the tracing of the a2-loop from the end of the A2 domain across the face of fVa, which contributes the majority of the reported contact surface with fXa and prothrombin. Even though the positioning of the a2-loop is physically problematic (potentiated by the breaking of a disulfide bond, followed by a *cis*-peptide configuration) and is completely unsupported by the map, its movement and its contacts are the main story of the paper. This bears a striking similarity to another cryo-EM structure, also published in *Blood* by the Di Cera group, of fV-short (8FDG, EMD-29011) [33]. The main point of the paper is the positioning of the unstructured loop C-terminal to the A2 domain (the short B-domain) that contains the a2-loop. This has been commented on extensively on PubPeer, where all reviewers express surprise that the map does not support the position of the loop when this comprises the main point of the paper. This is also a feature of the first-ever cryo-EM paper published by this group, also published in *Blood*, describing the cryo-EM structures of fVa and of fV (7KVE) [14]. As described earlier in this review, the position of the a2loop in 7KVE is not supported by the map and constitutes a main point of the paper.

The considerable effort of conducting and publishing this analysis was made so that researchers in the field of haemostasis will not waste time and money running experiments based on these erroneous results and so that the field will not consider the important issue of prothrombinase structure and function to be resolved. Unfortunately, the majority of biologists and biochemists do not have the tools or know-how to evaluate the veracity of crystal or cryo-EM structures and simply accept the reported findings. However, it has become trivial recently for non-experts to visualize maps and coordinates using programs such as Coot [22], and we encourage readers of this manuscript to evaluate 7TPP and the other structures mentioned here on their own. It is also evident that there are serious deficiencies in the way cryo-EM structures are validated and reviewed. A step in the right direction would be for journals to ensure that reviewers have access to coordinate and map files during the review process to assess the validity of claims, and that deposition of raw data, if requested, is a condition of publication. Publishers must also endeavour to make more avenues available for challenging questionable work, especially in the age of online-only contributions.

## Data Availability

Supplementary material is available online [34].
